# Solubility of recombinant Src homology 2 domains expressed in *E. coli* can be predicted by TANGO

**DOI:** 10.1186/1472-6750-14-3

**Published:** 2014-01-14

**Authors:** Thorny Cecilie Bie Andersen, Kjersti Lindsjø, Cecilie Dahl Hem, Lise Koll, Per Eugen Kristiansen, Lars Skjeldal, Amy H Andreotti, Anne Spurkland

**Affiliations:** 1 Department of Anatomy, Institute of Basal Medical Sciences, University of Oslo, Oslo, Norway; 2 Department of Biosciences, University of Oslo, Oslo, Norway; 3 Department of Chemistry, Biochemistry and Food science, Norwegian University of Life Sciences, Ås, Norway; 4 Department of Biochemistry, Biophysics and Molecular Biology, Iowa State University, Ames, Iowa, USA

**Keywords:** Bacterial inclusion bodies, Protein aggregation, Recombinant protein expression, SH2 domain, SH2D2A, Protein solubility

## Abstract

**Background:**

Signalling proteins often contain several well defined and conserved protein domains. Structural analyses of such domains by nuclear magnetic spectroscopy or X-ray crystallography may greatly inform the function of proteins. A limiting step is often the production of sufficient amounts of the recombinant protein. However, there is no particular way to predict whether a protein will be soluble when expressed in *E.coli.* Here we report our experience with expression of a Src homology 2 (SH2) domain.

**Results:**

The SH2 domain of the SH2D2A protein (or T cell specific adapter protein, TSAd) forms insoluble aggregates when expressed as various GST-fusion proteins in *Escherichia coli (E. coli)*. Alteration of the flanking sequences, or growth temperature influenced expression and solubility of TSAd-SH2, however overall yield of soluble protein remained low. The algorithm TANGO, which predicts amyloid fibril formation in eukaryotic cells, identified a hydrophobic sequence within the TSAd-SH2 domain with high propensity for beta-aggregation. Mutation to the corresponding amino acids of the related HSH2- (or ALX) SH2 domain increased the yield of soluble TSAd-SH2 domains. High beta-aggregation values predicted by TANGO correlated with low solubility of recombinant SH2 domains as reported in the literature.

**Conclusions:**

Solubility of recombinant proteins expressed in *E.coli* can be predicted by TANGO, an algorithm developed to determine the aggregation propensity of peptides. Targeted mutations representing corresponding amino acids in similar protein domains may increase solubility of recombinant proteins.

## Background

**Figure 1:**
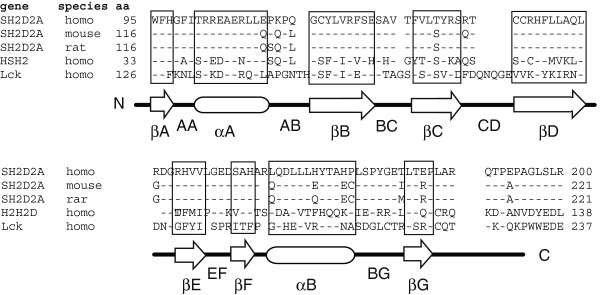
**Alignment of SH2 domains.** Alpha helices are indicated by open ovals and beta sheets are indicated by open arrows. “-“: position is identical to human SH2D2A sequence. “ “: gap in the alignment. Nomenclature for SH2 domain segments from [3].

Src homology 2 (SH2) are structurally conserved protein domains of approximately100 amino acids (aa). SH2 domains regulate numerous intracellular signal transduction events through interaction with tyrosine phosphorylated proteins [1]. Within the human genome, 110 distinct genes encode for 120 SH2 domains [2]. SH2 domains consist of an N-terminal alpha helix (αA) flanking a central five-stranded anti-parallel beta sheet (βB-F) followed by a second alpha helix (αB) and a C-terminal beta strand [3] (Figure 1). The five strands of the central beta sheet separate a conserved phosphotyrosine binding pocket from a more variable pocket that typically binds the third amino acid C-terminal to the phosphorylated tyrosine.

Detailed structural analyses have revealed specific and unique features of different SH2 domains suggesting that these domains, while ubiquitous and highly conserved, nevertheless exhibit unique specificity determining features. For example, NMR analysis of the SH2 domain of Inducible Tec Kinase (Itk) showed that its binding specificity is modulated by cis-trans isomerization of a single proline [4]. Structural analysis of the SH2D1A gene product, Slam associated protein (SAP), revealed the structural basis for a phosphotyrosine independent three-pronged binding mechanism of peptides to its SH2 domain [5]. Similarly, a disulphide bond in the SH2 domain of the C-terminal Src kinase (Csk) was found to be central for the kinase activity [6].

The function and specificity determining elements of many SH2 domain containing proteins, are still not well characterised. One of these is the T cell specific adapter protein (TSAd), encoded by the *SH2D2A* gene and expressed in activated T cells [7] and endothelial cells [8]. TSAd contains one SH2 domain, followed by a proline rich region and possesses ligands for SH2 and SH3 domains [9]. Hitherto only a few ligands for the human TSAd SH2 domain are known, including the phosphorylated vascular endothelial growth factor receptor 2 (VEGFR-2) [8,10], and the phosphorylated valocin containing protein (VCP) [11]. Recruitment of TSAd via SH2 domain binding to phosphorylated Y951 in the VEGFR2 receptor controls migration of [8] as well as permeability [12] of endothelial cells. Similarly, binding of TSAd via its SH2 domain to VCP is required for nuclear translocation of TSAd [11]. Preliminary results from our lab indicate that the TSAd-SH2 domain has additional ligands in activated T cells (Hem, unpublished observations). Given the interesting biology surrounding TSAd, and the role of SH2 domains in mediating important interactions in the context of cell signalling, we wanted to characterize the structure of TSAd SH2. However, when expressed as a GST-fusion protein in *E. coli*, the TSAd SH2 domain was found to be highly insoluble presenting a significant challenge to detailed structural characterization.

An algorithm for prediction of amyloid fibril formation in eukaryote cells, TANGO, has been developed [13], and it has been suggested that TANGO may also predict inclusion body formation in prokaryotic cells [14]. Here we report that TANGO predicts the solubility of recombinant SH2 domains expressed in *E.coli*, and that predictions made by TANGO allowed for targeted mutations that significantly improved the solubility of the recombinant protein.

## Results

### Low solubility of bacterially expressed human TSAd SH2 domain

**Figure 2:**
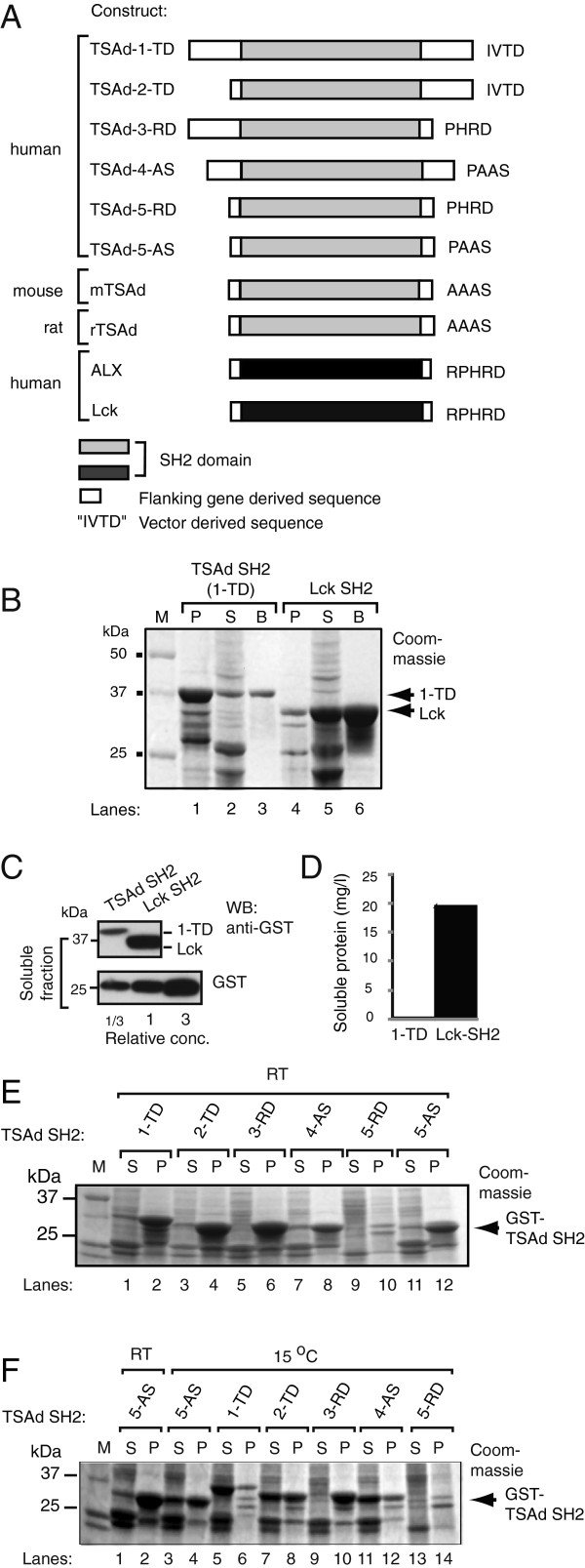
**Expression and solubility of GST-TSAd SH2 domains are influenced by flanking sequence and growth temperature. A**. Schematic overview of the GST-fusion SH2 constructs (given in Table 1) used in this study. **B**. GST-fusion SH2 constructs of TSAd (1-TD, TSAd-67-207-IVTD) and Lck (Lck-SH2, Lck-124-228-RPHRD) were expressed in *E.coli* at room temperature (RT). Equal volumes of resuspended pellet (P), soluble fraction (S) and glutathione beads **(B)** were separated by 10% SDS-PAGE. Proteins were visualised by Coomassie Brilliant Blue staining. **C**. Quantitation of the amount of soluble GST-SH2 proteins shown in **B**, by immunoblotting with anti-GST and comparison to defined amounts of GST. 5 μl of 2 ml TSAd-SH2 soluble fraction and 0,06 μl of 2 ml Lck-SH2 soluble fraction from 100 ml bacterial cultures were applied on the gel. GST = 1 represents a total amount of 0,11 μg GST applied on the gel. **D**. Quantitation of soluble GST-SH2 proteins using Image J analysis based on **C. E** and **F**. Yield of expression of the six GST-TSAd SH2 domain constructs in *E.coli* at RT or 15°C, respectively. Gels processed as in **B**. Constructs are indicated by their short names as listed in Table 1.

To perform biochemical and structural studies on the TSAd SH2 domain, we initially generated a GST-fusion construct (1-TD, Figure 2A) including the flanking amino acids 67–94 and 189–207. The resulting GST-TSAd SH2 domain was expressed mainly as insoluble inclusion bodies (compare lane 1 and 2, Figure 2B). In contrast, the GST-Lck SH2 protein containing only minimal gene-derived flanking sequence was primarily expressed in the soluble fraction (compare lane 4 and 5, Figure 2B). Although small amounts of recombinant GST-TSAd SH2 was present in the lysate, most of this soluble fraction could be captured on glutathione sepharose beads (lane 3, Figure 2B). Anti-GST-immunoblotting of equal amounts of the soluble fractions compared to a defined amount of purified, soluble GST (Figure 2C,i), revealed that the yield of the TSAd SH2 domain) was only 0,15 mg/l bacterial cell culture, while that of Lck-SH2 was estimated to be close to 20 mg/l bacterial culture (Figure 2D).

### Flanking sequences, growth temperature and genetic variation influence recombinant expression of TSAd SH2 domains

Having found that the protein yield of the TSAd-SH2 domain construct was only a fraction of that of the Lck-SH2 construct, additional constructs including different lengths of the sequence flanking the human TSAd SH2 domain and three different vector-encoded C-termini (Figure 2A and Table 1) were generated. When expressed at room temperature, the yield of these TSAd SH2 domain constructs varied considerably as judged by Commassie staining of SDS-gels. Compared to the TSAd-67-207-IVTD construct (1-TD), truncation of the sequence N- and/or C-terminal to the TSAd SH2 domain yielded approximately equal levels of expressed protein (Figure 2E, lanes 2, 4, 6 and 12). By contrast the TSAd-90-188-PHRD (5-RD) construct was not expressed at all (Figure 2E, lane 9 and 10). This construct only differed from TSAd-90-188-PAAS (5-AS) in its C-terminal vector-derived sequence, and from TSAd-67-188-PHRD (3-RD) in that the latter includes 37 aa N- terminal to the SH2 domain (Table 1).

**Table 1:** Overview of SH2 domain constructs included in this study

**Short name**	**Construct**	**Gene**	**Accession number**	**Species**	**Start aa**	**End aa**	**C-terminal vector derived sequence**	**Construct total length (aa)**
1-TD	TSAd-67-207-IVTD	*SH2D2A*	NP_003966	H sapiens	S67	S207	IVTD	145
2-TD	TSAd-90-207-IVTD	*SH2D2A*	NP_003966	H sapiens	G90	S207	IVTD	122
3-RD	TSAd-67-188-PHRD	*SH2D2A*	NP_003966	H sapiens	S67	R188	PHRD	126
4-AS	TSAd-81-193-PAAS	*SH2D2A*	NP_003966	H sapiens	T81	P193	PAAS	117
5-RD	TSAd-90-188-PHRD	*SH2D2A*	NP_003966	H sapiens	G90	R188	PHRD	103
5-AS	TSAd-90-188-PAAS	*SH2D2A*	NP_003966	H sapiens	G90	R188	PAAS	103
mTSAd	mTSAd-111-210-AAAS	*SH2D2A*	NP_001020742	M musculus	K111	R210	AAAS	104
rTSAd	rTSAd-111-209-AAAS	*SH2D2A*	NP_997488	R norvegicus	R111	R209	AAAS	103
ALX	ALX-31-126-RPHRD	*HSH2*	NP_116244	H sapiens	G30	R126	RPHRD	101
Lck	Lck-124-228-RPHRD	*Lck*	NP_005347	H sapiens	P124	K228	RPHRD	110

Growth temperature affects the solubility of recombinant proteins in *E.coli*[15]. Expression at 15°C resulted in higher yield of soluble TSAd SH2 domain from the TSAd-67-207-IVTD (1-TD), TSAd-90-207-IVTD (2-TD), TSAd-81-193-PAAS (4-AS) and TSAd-90-188-PAAS (5-AS) constructs, whereas solubility and expression of the TSAd-67-188-PHRD (5-RD) and TSAd-90-188-PHRD (3-RD) constructs remained unchanged (Figure 2F). We also tested whether the genetic variation inherent in species differences (Figure 1) affected expression of TSAd SH2 domains. Analysis of solubility and yield of identically cloned TSAd SH2 domains derived from three different species (constructs TSAd-90-188-PAAS (5-AS), mTSAd-111-210-AAAS (mTSAd) and rTSAd-111-210-AAAS (rTSAd), Table 1) revealed slightly improved solubility at 15°C of murine TSAd SH2 compared to human and rat (data not shown). Taken together, both gene- and vector-derived sequence influence the overall expression and solubility of recombinant TSAd-SH2 proteins. However, none of the conditions tested resulted in amounts of soluble recombinant TSAd SH2 domain sufficient for structural analysis.

### The TSAd SH2 domain contains a short peptide sequence promoting intermolecular beta-sheet aggregation

**Figure 3:**
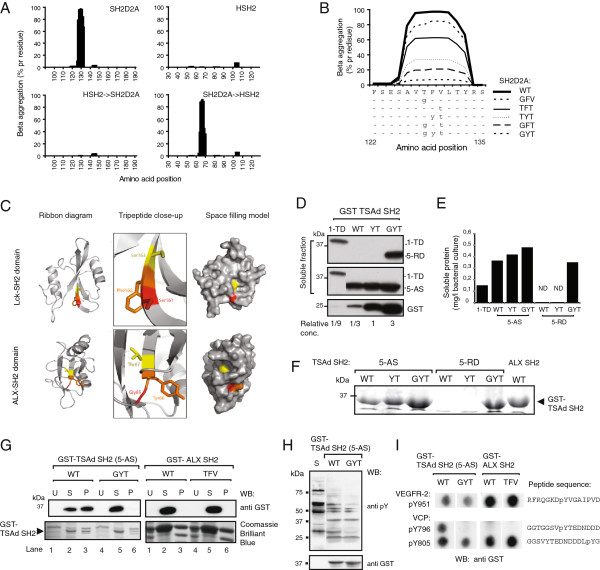
**Beta-aggregation prediction of the SH2D2A SH2 domain. A**. Amino acid positions of the indicated SH2 domains (X-axis) plotted against the corresponding beta-aggregating value (Y-axis). **B***.* Beta-aggregation values of amino acids 122–135 in wild type (WT) and *in silico* mutated SH2D2A encoding TSAd. **C**. Ribbon diagrams and space filling models of the Lck SH2 domain (PDB ID: 1BHH) and the ALX SH2 domain (PDB ID: 2CS0) showing the location of Ser-Phe-Ser (Lck) and Gly-Tyr-Thr (ALX) corresponding to the TSAd TFV sequence. Models show that the side chains of Ser 161 and Ser 163 in Lck SH2 and Gly65, Tyr66 and Thr67 of ALX SH2 are solvent accessible. **D**.-**F**. WT and mutated SH2 domains expressed in *E.coli* at 15°C. **D**. Equal amount (5 μl) of soluble fraction from a 100 ml bacterial culture was separated by SDS-page and immunoblotted with anti-GST antibodies for quantification of soluble recombinant protein. GST = 1 equals 0,11 μg purified GST protein. One representative experiment out of three is shown. **E**. Quantitation of soluble GST-SH2 proteins by Image J analysis based on D. ND; not detected. **F**. Soluble protein captured on glutathione beads were processed as in Fig. 2B. **G**. Solubility of WT and mutated TSAd SH2 (5-AS, TSAd-90-188-PAAS) (GYT) and ALX SH2 (TFV) expressed in *E.coli.* Uninduced *E.coli* (U), soluble (S) and pellet (P) fractions were processed as in Fig. 2B. Amount of soluble and insoluble SH2 domain visualized by anti-GST immunoblotting (upper panel). Total protein content in samples was visualized by Coomassie Brilliant Blue Staining (lower panel). **H**. CD3 stimulated Jurkat cell lysates (S) and proteins pulled down with the indicated SH2 domains were separated by SDS-page and immunoblotted. **I**. Peptide array with VEGFR-2 and VCP phosphotyrosine peptides probed with the indicated SH2 domains. Bound SH2 domains detected using anti-GST antibody.

In order to examine whether the TSAd SH2 domain has some intrinsic property limiting its solubility in prokaryotic cells [14], we took advantage of an algorithm for prediction of beta-aggregation stretches in proteins [13], TANGO (http://tango.crg.es). TANGO analysis of the human TSAd SH2 domain sequence (S90-R188) revealed that the βC strand of the human TSAd SH2 domain (Figure 1) harbours a nine aa sequence (SAVTFVLTY) with near 100% propensity for intermolecular beta-sheet aggregation (Figure 3A). Similar results were obtained for the murine and rat sequences, whereas no beta-aggregating regions were found in the Lck-SH2 domain, nor in the TSAd homologue ALX SH2 [2,16] (data not shown, and Figure 3A, upper right panel). *In silico* substitution of the TSAd sequence AVTFVLT to the corresponding ALX sequence HVGYTLS and vice versa, reduced the predicted beta-aggregation propensity of the TSAd SH2 domain to near zero, whereas the ALX SH2 domain attained beta-aggregation propensity similar to that of the native TSAd SH2 domain (Figure 3A, bottom panels). Further *in silico* replacements revealed that exchange of the TFV sequence in TSAd to the GYT sequence of ALX, resulted in a 90% reduction in the overall beta-aggregation propensity of TSAd SH2 (Figure 3B). As the structure of the TSAd-SH2 domain is not yet determined, we instead compared the structures of the Lck-SH2 domain and the ALX-SH2 domain to visualize the putative localisation of the TFV sequence in the SH2 domain structure (Figure 3C). The tripeptide region corresponding to the TSAd-SH2 TFV sequence (SFS and GYT respectively) is highlighted. As can be seen from the space filling models the Ser 161 and Ser 163 in Lck, and all three amino acids in the ALX-motif (Gly65, Tyr66 and Thr67), are solvent accessible. Given the structural conservation between SH2 domain folds, it is likely that at least part of the TFV sequence in TSAd-SH2 may be solvent exposed. When the TFV sequence from TSAd-SH2 was *in silico* replaced with the GYT sequence in the ALX-SH2 domain sequence, the TANGO algorithm predicted an increase in beta-aggregation propensity corresponding to the TFV sequence (Table 2). To directly test the predictions made by TANGO, we generated TFV → GYT and FV → YT mutants of the TSAd-90-188-PHRD and TSAd-90-188-PAAS constructs, and compared their expression to that of the ALX SH2 domain. These constructs were chosen, as they contained the shortest flanking sequences compared to the original TSAd 1-TD construct, and were thus better suited for the planned down stream analysis. The TFV → GYT mutation increased both yield and solubility of the TSAd SH2 domains (Additional file 1: Figure S1), most dramatically for the TSAd-90-188-PHRD construct, which was not expressed as soluble protein in its wild type form (Figure 2E and Figure 3D-F). As predicted by TANGO (Figure 3B and Table 2), the FV → YT replacement influenced solubility of the TSAd SH2 domain produced by TSAd-90-188-PAAS to a lesser extent, and had no effect in the TSAd-90-188-PHRD construct (Figure 3D-F). Compared to the original TSAd-SH2 construct (1-TD) which consistently yielded 0,15 mg/l bacterial culture (Figure 2D and 3E), the yield of soluble wild type TSAd-90-188-PAAS protein was doubled, and was further increased by 50% when mutated to GYT (Figure 3D), yielding close to 0,5 mg recombinant protein per liter bacterial cell culture. For the TSAd-90-188-PHRD protein mutated to GYT, the yield of soluble protein was doubled (i.e. 0,3 mg/l bacterial culture) compared to the original 1-TD construct (Figure 3D). When the reverse experiment was performed, replacing GYT sequence in the ALX SH2 domain with TFV, no adverse effect on solubility was observed (Figure 3G). An overview of the TANGO-predicted aggregation scores and yield of soluble protein for the various SH2 domains included in this study are given in Table 2. Thus, taken together TANGO predictions allowed for targeted mutations resulting in increased solubility of the TSAd SH2 domain in prokaryotic cells. However, the insertion of the TFV sequence alone into the ALX-SH2 domain was not enough to render this protein insoluble, indicating that TANGO will not always be able to predict solubility of recombinant proteins in *E.coli*.

**Table 2:** Overview of predicted TANGO aggregation score, production yield and pull-down activity for SH2 domain constructs included in this study

**Short name**	**Construct**	**TANGO predicted aggregation score**	**Production yield (mg/L bacterial culture)**	**pY-protein binding activity (pull-down)**
1-TD	TSAd-67-207-IVTD	793	0,15	ND
2-TD	TSAd-90-207-IVTD	774	ND	ND
3-RD	TSAd-67-188-PHRD	791	ND	ND
4-AS	TSAd-81-193-PAAS	771	ND	ND
5-RD	TSAd-90-188-PHRD	773	─	ND
5-RD YT	TSAd-90-188-PHRD	342	─	ND
5-RD GYT	TSAd-90-188-PHRD	103	0,35	ND
5-AS	TSAd-90-188-PHRD	772	0,37	+++
5-AS YT	TSAd-90-188-PAAS	339	0,42	ND
5-AS GYT	TSAd-90-188-PAAS	102	0,48	++
mTSAd	mTSAd-111-210-AAAS	745	ND	ND
rTSAd	rTSAd-111-209-AAAS	744	ND	+++*
ALX	ALX-31-126-RPHRD	63	ND	ND
ALX TFV	ALX-31-126-RPHRD	488	ND	ND
Lck	Lck-124-228-RPHRD	24	19,46	+++*

ND, not determined; ─, below detection limit; +++, strong signal; ++, signal.

*, data not shown.

### Mutant GST-TSAd SH2 domain binds to tyrosine phosphorylated proteins

Pull-down assays were performed to test whether the targeted mutations negatively influenced the function of the TSAd SH2 domain. Tyrosine phosphorylation of cellular proteins was induced in Jurkat T cells by anti-CD3 triggering. Lysates from stimulated cells were subjected to pull-down assay, using wild type and GYT-mutated GST-TSAd SH2 (TSAd-90-188-PAAS). Both constructs pulled down the same tyrosine phosphorylated proteins, although smaller amounts were pulled down using the GYT- mutated protein. However, some protein bands seemed to be more affected by the GYT mutation than others (Figure 3H). To further analyze the binding specificity of the mutated TSAd SH2 domain, peptide arrays representing phosphopeptides from VEGFR-2 and VCP, the two known TSAd SH2 domain ligands, were probed with wild type or mutated TSAd SH2 (TSAd-90-188-PAAS) domain. The results show that both TSAd wild type and GYT-mutated protein bind to VEGFR-2 phosphopeptide (pY951) and VCP (pY805), whereas binding to VCP pY796 was lost for the GYT-mutant (Figure 3I). The GYT → TFV replacement in ALX SH2 domain did not alter the specificity towards the selected phosphopeptides (Figure 3I). Taken together, the results show that although the mutant protein may have retained all or most of the assessed functions of the wild type protein, the specificity determining elements of the mutated protein domain may be altered. This should be carefully addressed when mutations are introduced for the purpose of increasing the solubility of the expressed protein.

### Low beta-aggregation values predict high solubility of recombinant SH2 domains

**Figure 4:**
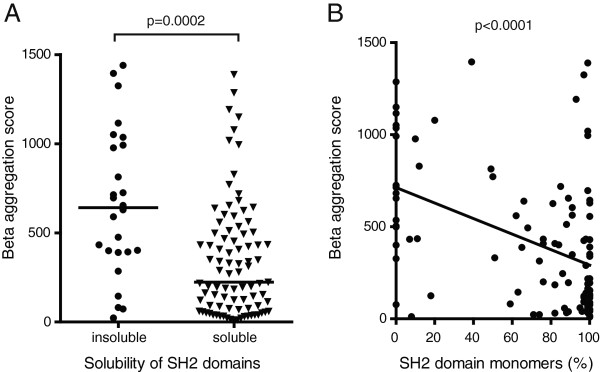
**Solubility of SH2 domains can be predicted by TANGO. A**. Median beta aggregation values (Y axis) of SH2 domains grouped according to solubility as reported by [19] (Mann-Whithney test p = 0.0002) **B**. Median beta aggregation values (+ SD) of SH2 domains plotted against corresponding reported fraction of monomers (%) [17]. Line is fitted with linear regression, using GraphPad Prism (R^2^ = 0.19, p < 0.0001).

Having found that TANGO could predict increased solubility of the TSAd-SH2 domain, we then asked whether beta-aggregation propensity is a common characteristic of SH2 domains. Most of the 120 SH2 domains encoded by the human genome have been expressed as recombinant His- [17] or GST-fusion proteins [18,19]. All available human SH2 domain sequences [2] were analysed by TANGO (Additional file 2: Table S1) and results were correlated to the recently published information on SH2 solubility. SH2 domains displaying low solubility had significantly higher beta-aggregation values than SH2 domains which were soluble [18] (Figure 4A) and [19] (data not shown). Similarly, the percentage of recombinant SH2 domains present as monomers (an indicator of protein solubility) as evaluated by analytical size exclusion chromatography [17], was inversely correlated with high beta-aggregation values (Figure 4B).

## Discussion

Expression of recombinant proteins is an important step forward for structural and functional analysis of entire proteins and isolated protein domains. The choice of tag may affect the solubility of the protein [20] and various parameters for successful expression of heterologous recombinant proteins have previously been reported [15,21]. However, there is no particular way to predict whether a protein will be soluble when expressed in *E.coli.* Having an interest in elucidating the function of the SH2 domain containing protein TSAd, we discovered that its SH2 domain was mainly insoluble when expressed in *E.coli*, as was also noted by others [17,18] but not all [19]. Although alteration in flanking sequence and growth temperature influenced expression and solubility of TSAd SH2, overall yield of soluble protein remained low.

Protein aggregation and amyloid fibril formation in degenerative diseases such as Alzheimer’s disease resembles the process of inclusion body formation in *E.coli*[14]. To our knowledge targeted mutation to relieve propensity for intermolecular beta-aggregation and increase solubility of recombinant proteins has not previously been reported, although the opposite experiment has been performed: de Groot and Ventura fused green fluorescent protein (GFP) to a mutated Aβ42 Alzheimer peptide to generate an aggregation prone reporter for monitoring protein quality in inclusion bodies by measuring GFP emission spectra [22]. Our results show that identification of aggregation prone proteins *in silico* allow for targeted mutations to increase solubility of the recombinant protein. Provided the overall structure and function of the recombinant protein are retained, this is a potentially efficient strategy to generate soluble recombinant protein for downstream purposes.

Aggregating peptide sequences are common within the hydrophobic core of globular proteins [23]. Accordingly, the beta-aggregating sequence identified by TANGO in the TSAd-SH2 domain (and also in many of the other SH2 domains with high beta-aggregating propensity (not shown)) was found in βC located in the core of the SH2 domain. Histidines, arginines or prolines are preferred immediately prior to, and after, aggregating sequences in the proteome. These gatekeeper amino acids may counteract beta-aggregating propensity, and may also serve as recognition sites for chaperones [24]. Although SH2 domains share overall structure, there is large sequence variability between individual domains of the family [2]. Interestingly, although most SH2 domains did not harbour aggregating βC sequences, the large majority still had histidine, arginine or proline flanking the βC sequence on each side (see alignment in [2]). This suggests that protein domain families may have evolved to resist the potential deleterious effects of beta-aggregating propensity of globular proteins.

## Conclusions

We have found that TANGO, an algorithm developed to predict amyloid fibril formation and aggregation of peptides, may also predict the solubility of recombinant SH2 domains expressed in *E.coli*. Predictions made by TANGO may allow for targeted mutations that significantly improve the solubility of the recombinant protein. Mutations introduced for the purpose of increasing the solubility of the expressed protein may alter specificity determining elements of the mutated protein domain.

## Methods

### Plasmid constructs

TSAd SH2, Lck SH2 and ALX SH2 encoding DNA were isolated from intact cDNA clones [9,16,25] by *in vitro* amplification, and cloned into the *BamHI/NotI* or *EcoR/NotI* site of the bacterial expression vector pGEX-6P-1 (GE Healthcare). The aa sequence alignment of the TSAd SH2 domains encoded by human, mouse and rat *SH2D2A* genes as well as the human Lck SH2 and ALX SH2 domains are shown in Figure 1. Additionally, Figure 1. includes the predicted secondary structures of these SH2 domains with nomenclature as defined in [3].The different constructs made are shown in Table 1 and are schematically represented in Figure 2A. Mutagenesis was performed using Quickchange (Clontech). All constructs were verified by sequencing.

### Protein expression

Glutathion S-transferase (GST) SH2 domain fusion proteins were made by isolating single clones of *E.coli* BL21-CodonPlus-RP (Stratagene,) transformed with appropriate cDNA constructs. Colonies were grown in LB medium at 37°C or in M9 minimal salt medium at 25°C to OD_600_ = 0.6-0.8, prior to addition of isopropyl-beta-D-thiogalactoside (IPTG) (Sigma-Aldrich) to a final concentration of 0.2 mM followed by incubation at room temperature or 15°C for 4–16 hours. Cultures were harvested by centrifugation, lysed in PBS with 0,5 mg/ml lysozyme followed by one freeze-thaw cycle (−80°C over night) and further lysis in GST-lysis buffer (1% Triton X-100, 1 mM DTT, 1 mM PMSF, 0,5 mg/ml lysozyme, 5 μg/ml DNAse, PBS pH 7.4) for ~16 h at 4˚C with agitation. Following centrifugation, soluble and insoluble (pellet) fractions were separated. The pellet was resuspended in an equal volume of PBS pH 7.4. Soluble and pellet fractions were analysed by SDS-PAGE followed by Coomassie Brilliant Blue staining or by western blotting using antibodies specific for GST (clone B14) (Santa Cruz Biotechnology, Santa Cruz, CA). Signals were developed using horse peroxidase-conjugated goat anti-mouse IgG (Jackson ImmunoResearch Laboratories, West Grove, PA) followed by Super Signal® west Pico Stable Peroxide Solution (Pierce, Rockford, IL).

### Protein purification

Protein was purified in bulk from lysates using Glutathione-Sepharose 4B beads (GE Healthcare) as described by the manufacturer. Beads were stored at 4°C in PBS pH 7.4. Expression level and purity of protein was examined by SDS-PAGE. Protein bands were directly visualised by staining of the gel with Coomassie Brilliant Blue.

### Antibodies*,* cell stimulation, and pulldown experiments

The following mAb were used; anti-human CD3ϵ (OKT3, American Type Culture collection, Manassas, VA), anti-GST (B-14,Santa Cruz Biotechnology, Santa Cruz, CA), anti-phosphotyrosine (anti-pY) (clone 4G10, Upstate Biotechnology, Lake Placid, NY), and horseradish peroxidase-conjugated goat-anti mouse IgG mAb (Sigma-Aldrich). Pull-down experiments using anti-CD3 stimulated Jurkat E6.1 cells (American Type Culture Collection) and GST-TSAd SH2 fusion proteins on glutathione sepharose beads were performed as previously described [9].

### Semi quantitative assessment of GST-fusion proteins

For semi-quantitative assessment of soluble GST-fusion proteins in bacterial lysate, GST was expressed from expression vectors without insert, purified in bulk as described above and then eluted from glutathione beads using glutathione as described by the manufacturer. The eluted protein was quantified using NanoDrop 2000c spectrophotometry (Thermo scientific, Wilmington, DE). A standard curve of 3 fold dilutions of the GST protein was included on the SDS-PAGE and the immunoblot signals from anti-GST (clone B14) (Santa Cruz Biotechnology, Santa Cruz, CA) were visually compared. Additionally, the amount of soluble protein was assessed by signal intensity meassurements using the image processing ImageJ software (http://rsb.info.nih.gov/ij/index.html). At least two independent membranes were examined for each quantitation.

### Peptide spot array analysis

Peptide arrays were synthesized on nitrocellulose membranes using a MultiPep automated peptide synthesizer (INTAVIS Bioanalytical Instruments AG, Germany) as described [26,27]. Membranes were probed with GST-tagged SH2 fusion proteins (80 μg/ml), and signals were detected using relevant antibodies followed by chemiluminescent detection.

### Prediction of protein aggregation

Prediction of aggregating regions of unfolded polypeptide chains were performed using the algorithm TANGO (http://tango.crg.es), using the default conditions pH 7.0, 25°C and 0.02 mM ionic strength [13,23,28].

## Abbreviations

aa: Amino acid; TSAd: T cell specific adapter protein; SH2: Src homology 2; mAb: Monoclonal antibody.

## Competing interests

The authors declare no financial or non-financial competing interests.

## Authors’ contributions

TCBA participated in the design of the study, did much of the laboratory work, analyzed the data and wrote the paper. KL did part of the laboratory work and analyzed the data. CDH did part of the laboratory work and analyzed the data, participated in the writing of the paper. LK did part of the laboratory work and analyzed the data. PEK participated in the design of the study and analyzed the data. LS participated in the design of the study. AHA participated in the design of the study and the analysis of data. ASP conceived of the study, analyzed data and participated in the writing of the paper. All authors have revised the manuscript critically for important intellectual content and have approved of the final version of the manuscript.

## Supplementary Material

Additional file 1: Figure S1 Yield and solubility of mutated TSAd-SH2 domains. WT and mutated SH2 domains as indicated were expressed in E.coli at 15°C. Soluble and pellet fractions were separated by 10% SDS-PAGE. Proteins were visualised by Coomassie Brilliant Blue staining. Click here for file

Additional file 2: Table S1 SH2 domains analysed by TANGO. Click here for file
