# Supplementary material for: Solubility of recombinant Src homology 2 domains expressed in E. coli can be predicted by TANGO
Source: BMC Biotechnol. 2014 Jan 14;14:3. doi: 10.1186/1472-6750-14-3 (PMC3922782; doi:10.1186/1472-6750-14-3)
Supplement: Additional file 1: Figure S1 — Yield and solubility of mutated TSAd-SH2 domains. WT and mutated SH2 domains as indicated were expressed in E.coli at 15°C. Soluble and pellet fractions were separated by 10% SDS-PAGE. Proteins were visualised by Coomassie Brilliant Blue staining. [file 1472-6750-14-3-S1.pdf]

Supplementary figure

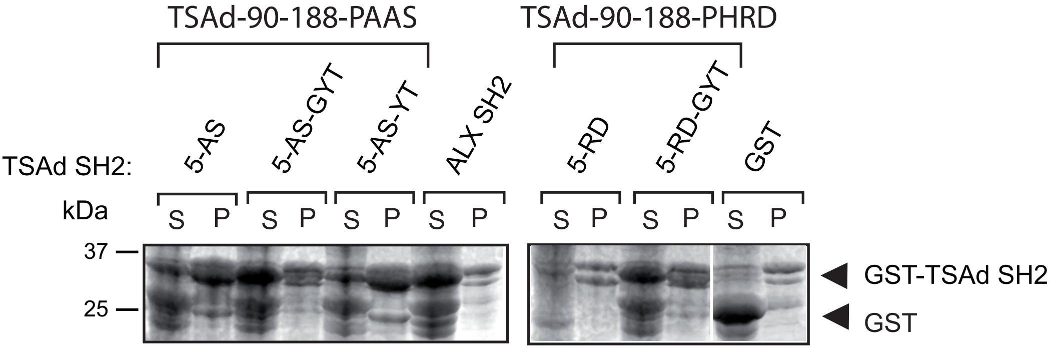

Figure legend:
WT and mutated SH2 domains as indicated were expressed in E.coli at 15 oC.
Soluble and pellet fractions were separated by 10% SDS-PAGE.
Proteins were visualised by Coomassie Brilliant Blue staining.
